# Efficient Reprogramming of Mouse Fibroblasts to Neuronal Cells including Dopaminergic Neurons

**DOI:** 10.1155/2014/957548

**Published:** 2014-06-01

**Authors:** Seung-ick Oh, Hang-soo Park, Insik Hwang, Han-kyul Park, Kyung-Ah Choi, Hyesun Jeong, Suhng Wook Kim, Sunghoi Hong

**Affiliations:** ^1^Laboratory of Stem Cell Biology, School of Biosystem and Biomedical Science, College of Health Science, Korea University, Jeongneung-dong, Seongbuk-gu, Seoul 136-703, Republic of Korea; ^2^Department of Biomedical Science, College of Health Science, Korea University, Jeongneung-dong, Seongbuk-gu, Seoul 136-703, Republic of Korea; ^3^Department of Health Science, College of Health Science, Korea University, Jeongneung-dong, Seongbuk-gu, Seoul 136-703, Republic of Korea

## Abstract

Somatic cells were directly converted to functional neurons through the use of a combination of transcription factors, including Ascl1, Brn2, and Myt1l. However, a major limitation is the lack of a reliable source of cell-replacement therapy for neurological diseases. Here, we show that a combination of the transcription factors Ascl1 and Nurr1 (AN) and neurotrophic factors including SHH and FGF8b directly reprogrammed embryonic mouse fibroblasts to induced neuronal (iN) cells: pan-neuronal cells and dopaminergic (DA) neurons under our systematic cell culture conditions. Reprogrammed cells showed the morphological properties of neuronal cells. Additionally, cells were analyzed using various markers, including Tuj1 and Map2 for neuronal cells and Lmx1a, Th, Aadc and Vmat2 for DA neurons in our immunostaining and reverse transcription (RT)-PCR experiments. We found that a combination of transcription factors and neurotrophic factors could directly reprogram fibroblasts to neuronal cells including DA neurons. Various types of reprogrammed cells are promising cell sources for cell-based therapy of neurological disorders like Parkinson's disease and spinal cord injury.

## 1. Introduction 

Cellular reprogramming by which somatic cells can be converted to induced pluripotent stem cells (iPSCs) and subsequently differentiated into mature cells is a breakthrough for disease modeling and cell-based therapy [1–4]. However, major limitations, such as low reprogramming efficiency and lengthy procedures, restrict the use of iPSCs [2, 5–7]. Moreover, clinical applications require subsequent redifferentiation into a specific cell type, and undifferentiated iPSCs may become tumorigenic by incomplete differentiation of iPSCs. Recently, it was shown that combined expression of defined factors could convert somatic cells into other somatic cell types such as brown fat [8], cardiomyocytes [9], hepatocyte-like cells [10, 11], hematopoietic progenitors [12], neural progenitors or neural precursor cells [13], neural stem cells [14, 15], glutamatergic neurons or GABAergic neurons [16], motor neurons [17], and neurons or dopaminergic (DA) neurons [18, 19]. Reprogrammed cells that do not pass through the pluripotent state may not be tumorigenic and may serve as a potential alternative to iPSCs for generating patient- and/or disease-specific neurons. However, published reprogramming protocols involve different combinations of various transcription factors to convert iPSCs into other mature cell types, making it difficult to generate a desired cell type.

Here, we showed that mouse embryonic fibroblasts could be directly reprogrammed into pan-neurons and DA neurons using a combination of the Ascl1 and Nurr1 transcription factors and various neurotrophic factors under our systematic cell culture conditions. However, our approach should be further optimized for use as a cell source for cell-based therapy to treat neurological disorders such as Parkinson's disease.

## 2. Materials and Methods

### 2.1. Cell Culture

MEFs were isolated and cultured as described previously [18] from embryonic day (E) 14.5 wild-type BALB/c mice embryos. Mouse experiments were approved by the Institutional Animal Care and Use Committee of Korea University (KUIACUC-2012-111) and were performed in accordance with government and institutional guideline and regulations. Briefly, MEFs were expanded up to passage 2 in an MEF medium consisting of DMEM containing 10% FBS, 1% NEAA, and 1% penicillin/streptomycin (all from Gibco, Grand Island, NY, USA) at 37°C, 5% CO_2_ in 95% humidity. At passage number 2, the MEF phenotype was confirmed by immunocytochemical analysis with a positive marker (vimentin) and negative markers (Sox1, Nestin, or Tuj1).

### 2.2. Retroviral Vectors Construction, Production, and Titration

Human Nurr1 cDNAs were amplified with primers for each gene using high-fidelity cloned* Pfu* DNA polymerase (Stratagene, La Jolla, CA, USA) and subcloned into the* Eco*RV site of the vector pUC19. Retroviral vectors expressing Nurr1 were constructed by inserting the respective cDNA derived from pUC19 into the monocistronic retroviral vector pCL. Retroviral vectors expressing Ascl1 were gifts from CH Park (Department of Microbiology, Hanyang University, Seoul, Korea). Retroviral vectors were introduced into the retrovirus packaging cell line 293 GPG by transient transfection with Lipofectamine 2000 (Invitrogen, Carlsbad, CA, USA). Supernatants were, respectively, collected each day for two weeks and stored in a deep freezer at −80°C [20]. Mouse embryonic fibroblasts (MEFs) (5 × 10^4^ cells/well) were transduced with viruses at multiplicity of infection (MOI) of 10 in the presence of polybrene (2 *μ*g/mL) for 18 h at passage 3. At 2 days after transduction, the transduced cells were seeded onto Cellstart-coated (Invitrogen) 24-well (5 × 10^4^ cells/well) or 6-well (2.5 × 10^5^ cells/well) plates and were cultured for 1 day in Dulbecco's modified Eagle medium (DMEM) containing 10% fetal bovine serum (FBS) and 1% penicillin/streptomycin. The next day, the medium was replaced with DMEM/F12-based neuronal medium (NM) supplemented with 1% ITS, 1% N2 supplement, 1% B27, 1% ascorbic acid, and 1% penicillin/streptomycin. The medium was replaced every other day.

### 2.3. Neuronal Precursor Cells

To induce neuronal precursor (NP) cells, the MEF medium was replaced with NM containing 20 ng/mL bFGF and 20 ng/mL EGF (R&D Systems, Minneapolis, MN, USA) at 3 days after transduction. MEF cells were cultured for an additional 17 days, and NP-like cells were analyzed by immunofluorescence and semiquantitative reverse transcription (RT)-RCR.

### 2.4. Neurons

To induce neurons, the medium was replaced and cultured with NM containing 20 ng/mL bFGF and 20 ng/mL EGF (R&D Systems) for 12 days at 3 days after transduction. The NM was changed to bFGF-/EGF-free NM for a further 16 days. Neuron-like cells were analyzed by immunofluorescence and semiquantitative RT-PCR.

### 2.5. DA Neurons

To induce DA neurons, the medium was replaced with NM containing 20 ng/mL bFGF and 20 ng/mL EGF at 3 days after transduction for 3 days. Next, 200 ng/mL Shh and 100 ng/mL FGF8b (R&D Systems) were added to the medium and the cells were cultured for an additional 9 days. The medium was changed with NM supplemented with only both Shh (200 ng/mL) and FGF8b (100 ng/mL), and then cells were cultured for an additional 10 days. DA neuron-like cells were induced by withdrawing both Shh and FGF8b from NM. Over the next 25 days, reprogrammed cells began to change to neuron-like cell morphology. DA neuron-like cells were analyzed by immunofluorescence and semiquantitative RT-PCR.

### 2.6. Immunofluorescence Assay

Cells were fixed in 4% paraformaldehyde and were stained with the following primary antibodies: rabbit *α*-rat Nurr1 (sc-991; 1 : 200; Santa Cruz Biotechnology, Santa Cruz, CA, USA); goat *α*-human Asc1 (sc-13219; 1 : 200; Santa Cruz Biotechnology); mouse *α*-human vimentin (V5255; 1 : 200; Sigma, St. Louis, MO, USA); rabbit *α*-mouse Sox1 (ab22572; 1 : 200; Abcam, Cambridge, UK); mouse *α*-human Nestin (MAB5326; 1 : 200; Millipore, Billerica, MA, USA); rabbit *α*-rat Tuj1 (MRB-435p; 1 : 1000; Covance, Princeton, NJ, USA); mouse *α*-human MAP2 (M2320; 1 : 200; Sigma, St. Louis, MO, USA); rabbit *α*-rat TH (AB152; 1 : 200; Millipore). Appropriate Alexa Fluor 488 or 594 F (ab′)2 fragment of goat *α*-rabbit or *α*-mouse IgG (H+L) antibodies or Alexa Fluor 555 goat *α*-rabbit IgG (H+L) or Alexa Fluor 594 F (ab′)2 fragment of rabbit *α*-goat IgG (H+L) (A-11070, A-11020 or A-21428, A-21223; 1 : 2000; Invitrogen) and DAPI (1 *μ*g/mL) counterstain were used for visualization. Fluorescence images were obtained using fluorescence microscopy (Evos, Amgmicro, Life Technologies, Carlsbad, CA, USA) and confocal laser scanning microscopy (Fluoview100, Olympus, Tokyo, Japan).

### 2.7. RT-PCR

Total RNA from cells of* in vitro* differentiation was prepared using Trizol Reagent (Invitrogen) followed by treatment with DNase I (Ambion, Austin, TX, USA). Two *μ*g of total RNA was reverse-transcribed into cDNA using oligo (dT) primers, according to the SuperScript III Reverse Transcriptase Kit (Invitrogen). The cDNA was then analyzed by semiquantitative RT-PCR using the neuronal gene primers. Relative expression of mRNAs was assessed by normalizing levels of cDNA to the signal from glyceraldehyde-3-phosphate dehydrogenase (GAPDH) mRNA. RT-PCR reactions were carried out on an ABI2720 Thermal Cycler (Applied Biosystems, Foster City, CA, USA) in final volumes of 30 *μ*L containing 1x reaction buffer (50 mM KCl, 2 mM MgCl_2_, and 25 mM TAPS pH 9.3), 0.2 mM dNTP, 0.5 *μ*M of each primer, and 1.25 units of TaKaRa Ex Taq (TaKaRa Biotechnology Co., Ltd., Shiga, Japan). Primer sequences, annealing temperatures, cycle numbers, and amplicon size are shown in Supplementary Table  1 available online at http://dx.doi.org/10.1155/2014/957548.

### 2.8. Statistical Analysis

Data was analyzed by using SPSS 12.0 software. All results were evaluated by a two-tailed Student's paired *t*-test and are shown between group mean ± SD from 3 independent experiments. *P* < 0.01 (∗) was considered statistically significant.

## 3. Results

### 3.1. Reprogramming of MEF Cells into Neuronal and Glial Cells by Ascl1 and Nurr1

**Figure 1:**
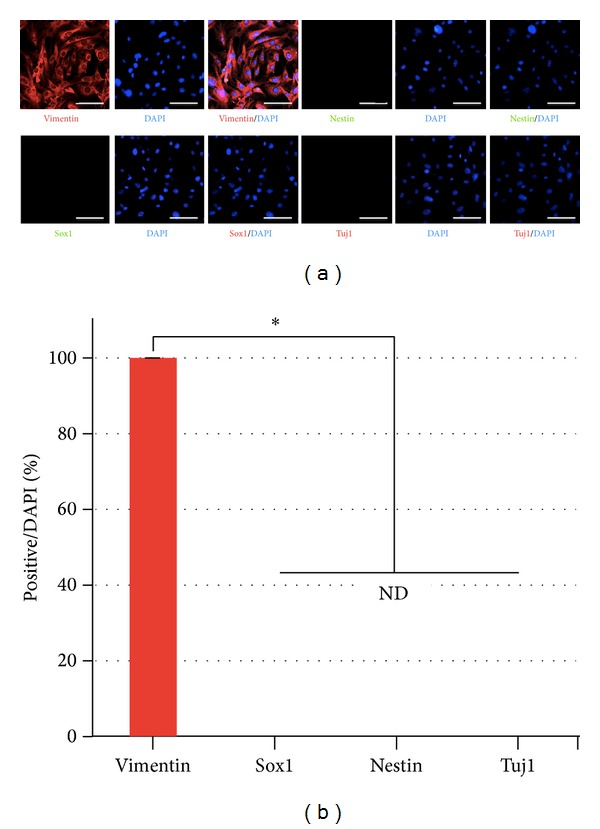
Isolation, characterization, and conversion into neural lineage of MEF cells. ((a), (b)) MEF cells did not express the neuronal markers but highly expressed the fibroblast marker. Magnification ×200; scale bar is 50 *μ*m. Single asterisk is *P* < 0.01.

For the direct conversion of somatic cells into neuronal lineage cells, we first prepared mouse embryonic fibroblasts (MEFs) by removing spinal cord parts from the mouse fetus on embryonic day 14.5 (E14.5). Then, we cultured the MEF in a Petri dish and checked the cells with immunostaining using anti-vimentin antibody as a fibroblast marker or anti-Nestin, anti-Sox1, and anti-Tuj1 antibodies as neural and pan-neuronal markers, respectively. We confirmed that our cultured MEF cells were uniformly positive against anti-vimentin but were negative against anti-Nestin, -Sox1, and -Tuj1 antibodies (Figures 1(a) and 1(b)). Next, MEF cells were infected with retroviral vectors containing Ascl1 and Nurr1, and then cultured for 25 to 30 days in neuronal medium (NM), which contained DMEM/F12 culture media supplemented with insulin/transferrin/selenium (ITS), N2, B27, and ascorbic acid (AA).

### 3.2. Induction of Neural Precursor (NP) Cells from Fibroblasts

**Figure 2:**
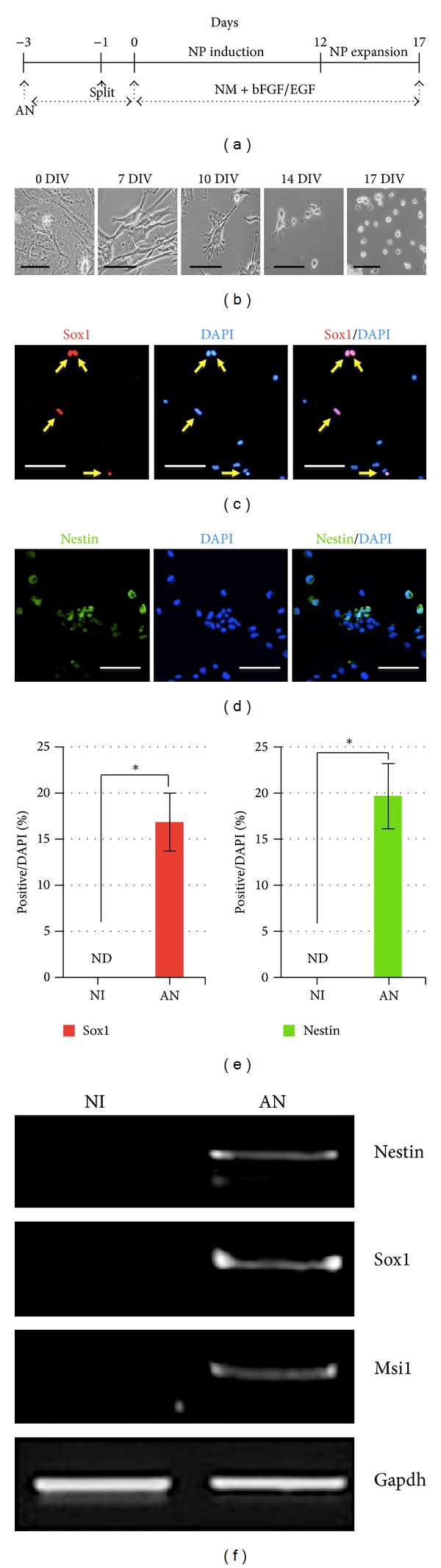
Direct conversion of MEF cells into neural precursor (NP) cells. (a) A schematic diagram for the generation of MEF-derived NP cells. (b) Representative phase-contrast images. Magnification ×200; scale bar is 100 *μ*m. ((c), (d)) Representative immunofluorescence image of differentiated MEF cells stained with Nestin and Sox1 antibodies. Yellow arrows on the above micrograph indicate Sox1-stained cells. Magnification ×200; scale bar is 50 *μ*m. (e) Reprogramming efficiency. Sox1-positive (red bar) and Nestin-positive (green bar) cells per DAPI-positive cells. Single asterisk is *P* < 0.01. (f) mRNA expression of neural precursor marker genes Nestin, Sox1, and Msi1 by RT-PCR.

Since the reprogramming potentials of MEF cells to neuronal and glial cells were demonstrated in our experiments, we examined whether MEF cells could be converted into neural precursor (NP) cells. Isolated MEF cells were maintained in serum-containing medium and were infected with the retroviral vectors containing Ascl1 and Nurr1. Three days after infection, infected cells were transferred onto a coated dish for 1 day and then cultured in NM containing 20 ng/mL bFGF and 20 ng/mL EGF for more than 17 days (Figure 2(a)). Interestingly, morphological changes began to appear at 7 days after induction, and the morphology of NP cells was clear by approximately 14 to 17 days after induction, with cells showing a large nucleus, narrow cytoplasm, and round shape (Figure 2(b)). At 21 days after induction, potential NP cells were analyzed for neural precursor markers Sox1, Nestin, and Musasi1. NP-like cells were clearly immunoreactive against Sox1 and Nestin antibodies in the nucleus and cytoplasm, respectively (Figures 2(c) and 2(d)). Sox1- and Nestin-positive cells accounted for approximately 16.82 ± 3.17% and 19.68 ± 3.51% out of total cells, respectively (Figure 2(e)). In addition, RT-PCR experiments showed that NP-like cells showed distinct expression of the neural precursor marker genes Nestin, Sox1, and Musasi1 (Figure 2(f)). Next, we examined whether NP-like cells could directly differentiate into neuronal and glial cells. For differentiation, NP-like cells were cultured for an additional 14 days in NM media without bFGF and EGF. However, NP-like cells were not differentiated into either neurons or glial cells. These results indicate that the Ascl1 and Nurr1 factors directly reprogram the somatic cells into neuronal cells without a NP-like cell stage, while NP-like cells appeared to be maintained for a period of time.

### 3.3. Direct Conversion of Fibroblasts to Pan-Neuronal Cells

**Figure 3:**
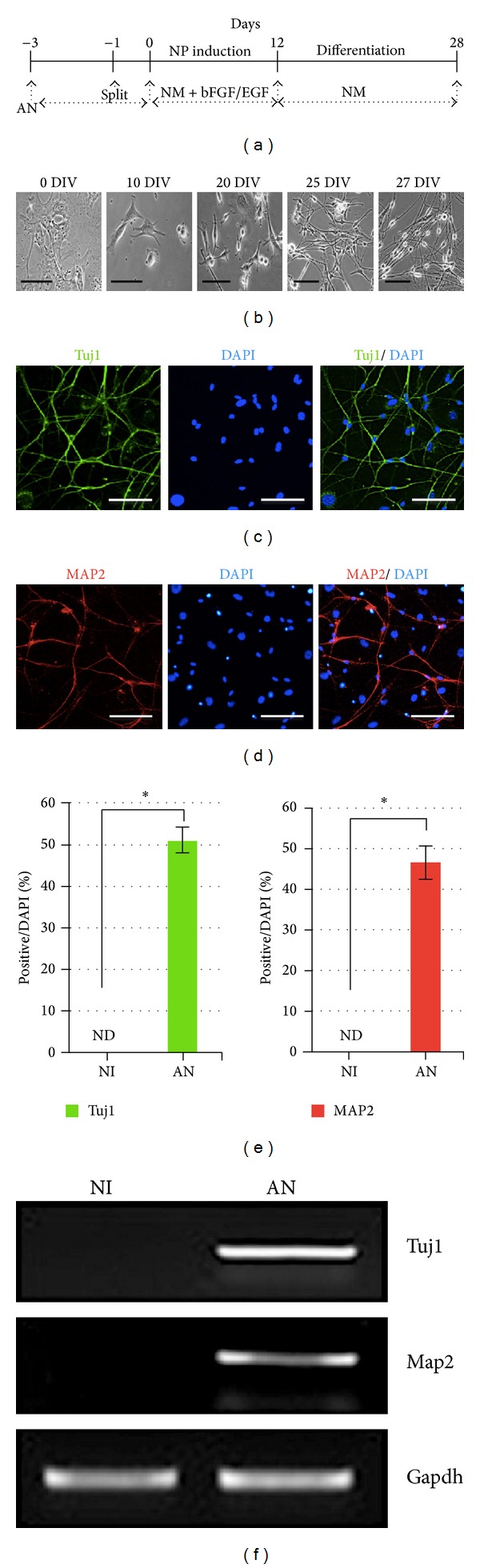
Direct conversion of MEF cells into neurons. (a) A schematic diagram of the generation of MEF-derived neurons. (b) Representative phase-contrast images of neuron-like cells showing bipolar projections. Magnification ×200; scale bar is 100 *μ*m. ((c), (d)) Representative immunofluorescence image of differentiated MEF cells stained with Tuj1 and MAP2 antibodies. Magnification ×200; scale bar is 50 *μ*m. (e) Reprogramming efficiency of the MEF cells into neuronal cells. Tuj1-positive (green bar) and MAP2-positive (red bar) cells per DAPI-positive cells. Single asterisk is *P* < 0.01. (f) mRNA expression of neuronal cell marker genes Tuj1 and Map2 by RT-PCR.

Since we confirmed that MEF cells were converted into neuronal cells in our culture system as occurred in Wernig's group [18], we examined whether MEFs could be directly converted into neurons. MEF cells that were transduced with retroviral vectors containing Ascl1 and Nurr1 were induced to neuronal cells in NM containing 20 ng/mL bFGF and 20 ng/mL EGF for 12 days. Next, differentiation of transduced cells was induced by withdrawing bFGF and EGF for a further 16 days or more (Figure 3(a)). The cells showed a neuronal cell type morphology, exhibiting neurite-like outgrowth. Neurites were extensively grown and branched out to form neural connections with other cells, as shown in a typical neuronal cell type, at days 25 to 27 (Figure 3(b)). Reprogrammed cells were stained with the pan-neuronal marker Tuj1 and the mature neuronal marker MAP2. Tuj1-positive cells were approximately 51 ± 3.02% and MAP2-positive cells were approximately 46 ± 4.01% of total cells by our immunofluorescence assay (Figures 3(c) and 3(d)). In addition, the neuronal genes Tuj1 and MAP2 were clearly expressed in the reprogrammed cells, as shown in our RT-PCR experiments (Figure 3(e)). These results indicate that the MEF cells were successfully and efficiently converted to neuronal cell type in our culture systems using Ascl1 and Nurr1 factors.

### 3.4. Direct Conversion of Fibroblasts to DA Neurons

**Figure 4:**
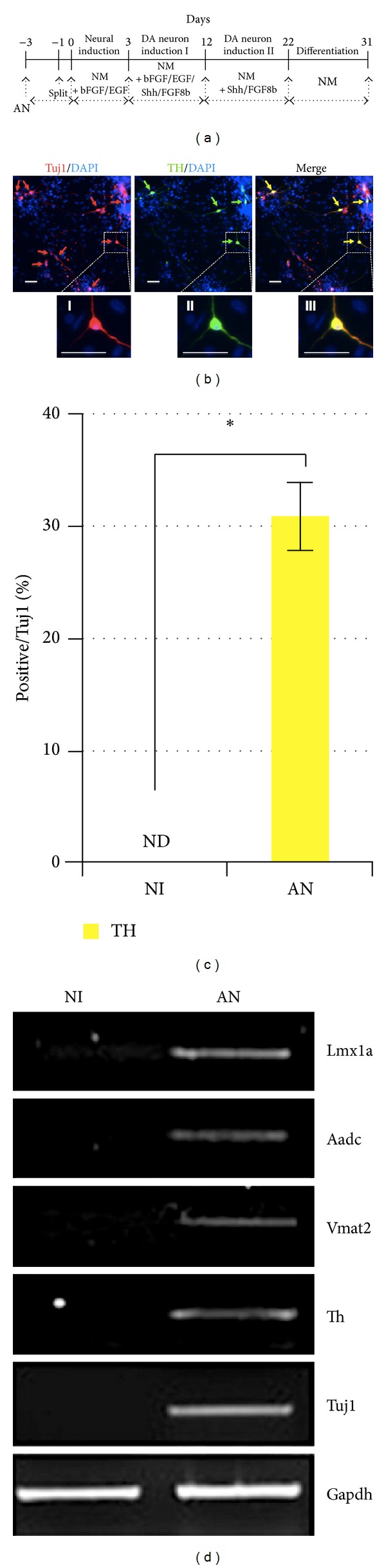
Direct conversion of MEF cells into dopaminergic (DA) neurons. (a) Schematic diagram of the generation of MEF-derived DA neurons. (b) Representative immunofluorescence of the DA neurons stained with Tuj1 and TH antibodies. A yellow arrow indicates DA neurons. Magnification ×200; scale bar is 50 *μ*m. (I, II, and III) Higher magnification insert of B. (c) Reprogramming efficiency of the MEF cells into DA neuron-like cells. Single asterisk is *P* < 0.01. (d) mRNA expression of various DA neuronal marker genes by RT-PCR.

We next examined whether MEF cells could be directly converted to DA neurons by Ascl1 and Nurr1. MEF cells were transduced with retroviral vectors containing Ascl1 and Nurr1. Transduced cells were cultured in NM containing 20 ng/mL bFGF and 20 ng/mL EGF for 3 days. At 3 days after neural induction, 200 ng/mL Shh and 100 ng/mL FGF8b were added to NM for 9 days, and the cells were cultured in NM containing 200 ng/mL Shh and 100 ng/mL FGF8b in the absence of bFGF and EGF. DA neurons were induced for 9 days by withdrawing Shh and FGF8b (Figure 4(a)). Cells were morphologically changed to neuron-like cells, which were positive against tyrosine hydroxylase (TH) and Tuj1 antibodies (Figure 4(b)). The efficiency of TH+ cells out of Tuj1+ cells was approximately 33 ± 0.62% (Figure 4(c)). Expression of Lmx1a, Aadc, Vmat2, Th, and Tuj1 genes was clearly detected by our RT-PCR experiments (Figure 4(d)). These results indicate that the conversion of MEF cells into a DA neuronal cell type can be successfully achieved under our systematic cell culture conditions, suggesting that MEF cells could be directly reprogrammed into DA neuronal cells by Ascl1 and Nurr1.

## 4. Discussion

Neuronal and glial cells, including neural precursor cells, are a promising cell source of cell-based therapy for neurological disorders [21]. It is also possible to directly generate a desired cell type from either skin or other somatic cells [8–19, 22, 23]. In this study, we demonstrated that a defined set of transcription factors, Ascl1 and Nurr1, can directly convert embryonic mouse fibroblasts into neuronal cells including DA neurons using our established culture systems supplemented with various neurotrophic factors.

A major concern when using embryonic fibroblasts for conversion is that contamination of neural precursors or glial and neuronal cells may be present in the starting materials. To exclude this possibility, we confirmed that there were only fibroblasts in our cell cultures by using the fibroblast marker vimentin and various other neural markers, including Sox1, Nestin, and Tuj1. We detected no Nestin- or Sox1- or Tuj1-positive cells (Figure 1(a)), suggesting no contamination of neural cells in our fibroblast population. When we first directly converted the fibroblasts into neural precursors by using Ascl1 and Nurr1 transcription factors, reprogrammed cells were positive against neural markers and clearly expressed neural marker genes compared to fibroblasts, as shown in Figure 2. However, NP cells did not proliferate during the cell expansion step. These results suggest that NP cells might be directly generated from MEF cells, but they seem to be maintained only for a short period of time during induction of MEFs to NP cells. Our reprogramming protocol should be further optimized for clinical applications.

In addition, we also directly reprogrammed fibroblasts to Tuj1-positive neurons using Ascl1 and Nurr1 in our cell culture conditions supplemented with various cytokines. Interestingly, we found that fibroblasts were directly converted into neurons with high reprogramming efficiency, which was approximately 51.0 ± 3.02%, suggesting that our reprogramming protocols can be used to generate a cell source of cell-based therapy for neurological disorders. Moreover, we directly reprogrammed fibroblasts into TH-positive DA neurons using two Ascl1 and Nurr1 factors in our cell culture systems supplemented with SHH and FGF8b. The reprogramming efficiency of TH+ cells was relatively high, which was approximately 33 ± 0.62%. These results suggest that the reprogramming protocol for generating reprogrammed DA neurons may be used as an alternative cell source of cell-based therapy for clinical application of Parkinson's disease in the future.

Nurr1 is a member of the nuclear receptor family regulating genes that are involved in the induction and maintenance of DA neurons [24–27]. Recently, Caiazzo et al. converted mouse and human fibroblasts into functional DA neurons using the 3 factors Ascl1, Nurr1, and Lmx1a with relatively high efficiency (~18%) [28]. They also showed that the factors Ascl1 and Nurr1 directly reprogrammed fibroblasts into DA neuronal cells with low reprogramming efficiency (~8%). In addition, neurotrophic factors such as SHH and FGF8 have been known to be critical for the specification and development of a neuronal subtype [29, 30]. Recently, Castro et al. reported that Ascl1 can directly control the specification of neural progenitors, as well as the later steps of neuronal differentiation and neurite outgrowth, in the embryonic brain and in neural stem cell cultures [31]. These reports support the fact that Ascl1 and Nurr1 are sufficient for reprogramming somatic cells into neuronal cells under our culture conditions using various neurotrophic factors. Importantly, our approach prevents development of tumorigenicity of ESCs or iPSCs when they were transplanted in an undifferentiated state. Therefore, reprogramming of pan-neuronal and DA neuronal cells can be used for cell-based therapy to treat neurological disorders such as Parkinson's disease and may also be invaluable for mechanistic studies and drug screening.

## 5. Conclusions

This study demonstrates that mouse embryonic fibroblasts were directly reprogrammed into pan-neurons and DA neurons using a combination of the Ascl1 and Nurr1 transcription factors and various neurotrophic factors under our systematic cell culture conditions. In particular, the reprogramming efficiency of pan-neuronal cells (Tuj1+) and DA neurons (TH+) was approximately 51.0 ± 3.02% and 33 ± 0.62%, respectively, indicating relatively high. However, it should be further studied whether the reprogrammed neuronal cells are functional.

## Supplementary Material

Supplementary Table 1: Sequences for RT-PCR primers used in this study
