# Supplementary material for: Efficient Reprogramming of Mouse Fibroblasts to Neuronal Cells including Dopaminergic Neurons
Source: ScientificWorldJournal. 2014 Jun 1;2014:957548. doi: 10.1155/2014/957548 (PMC4058809; doi:10.1155/2014/957548)
Supplement: Supplementary file 1 — Supplementary Table 1: Sequences for RT-PCR primers used in this study [file 957548.f1.pdf]

Supplementary Table 1. Sequences for RT-PCR primers used in this study

Gene	Annealing temperature, Cycle number	Forward primer (5′→3′)	Reverse primer (5′→3′)	Amplicon size
Aadc	58°C, 35 cycles	CCTACTGGCTGCTCGGACTAA	GCGTACCAGTGACTCAAACTC	715
Gapdh	56°C, 28 cycles	TGACATCAAGAAGGTGGTGAAGC	CCCTGTTGCTGTAGCCGTATTC	203
Lmx1a	60°C, 32 cycles	CTCACCCCACCCCAGATGCCT	CTCCCTCCCCAGCCCACCTCT	316
Map2	58°C, 32 cycles	AGCCGCAACGCCAATGGATT	TTTGTTCTGAGGCTGGCGAT	313
Msi1	58°C, 32 cycles	CACGACCCCTGCAAGATGTTC	CCATCTTAGGCTGTGCTCTTCGA	265
Nestin	58°C, 32 cycles	GGAGAGTCGCTTAGAGGTGC	TCAGGAAAGCCAAGAGAAGC	327
Sox1	58°C, 35 cycles	GCCCAGGAAAACCCCAAGATG	CCGTTAGCCCAGCCGTTGAC	362
Th	55°C, 30 cycles	AGGGATGGAAATGCTGTTCTCA	ACCAGGTGGTGACACTTATCCAA	314
Tuj1	60°C, 32 cycles	TCAGCGATGAGCACGGCATA	CACTCTTTCCGCACGACATC	305
Vmat2	58°C, 35 cycles	ATCCAGACTGCCAGGCCAGCG	CTCCATCCAAGAGCACCAAGG	622
